# Methods for Surgical Targeting of the STN in Early-Stage Parkinson’s Disease

**DOI:** 10.3389/fneur.2014.00025

**Published:** 2014-03-19

**Authors:** Corrie R. Camalier, Peter E. Konrad, Chandler E. Gill, Chris Kao, Michael R. Remple, Hana M. Nasr, Thomas L. Davis, Peter Hedera, Fenna T. Phibbs, Anna L. Molinari, Joseph S. Neimat, David Charles

**Affiliations:** ^1^Department of Neurosurgery, Vanderbilt University Medical Center, Nashville, TN, USA; ^2^Stritch School of Medicine, Loyola University Chicago, Chicago, IL, USA; ^3^Department of Neurology, Vanderbilt University Medical Center, Nashville, TN, USA

**Keywords:** Parkinson’s disease, subthalamic nucleus, surgical targeting, early stage, neurosurgery

## Abstract

Patients with Parkinson’s disease (PD) experience progressive neurological decline, and future interventional therapies are thought to show most promise in early stages of the disease. There is much interest in therapies that target the subthalamic nucleus (STN) with surgical access. While locating STN in advanced disease patients (Hoehn–Yahr Stage III or IV) is well understood and routinely performed at many centers in the context of deep brain stimulation surgery, the ability to identify this nucleus in early-stage patients has not previously been explored in a sizeable cohort. We report surgical methods used to target the STN in 15 patients with early PD (Hoehn–Yahr Stage II), using a combination of image guided surgery, microelectrode recordings, and clinical responses to macrostimulation of the region surrounding the STN. Measures of electrophysiology (firing rates and root mean squared activity) have previously been found to be lower than in later-stage patients, however, the patterns of electrophysiology seen and dopamimetic macrostimulation effects are qualitatively similar to those seen in advanced stages. Our experience with surgical implantation of Parkinson’s patients with minimal motor symptoms suggest that it remains possible to accurately target the STN in early-stage PD using traditional methods.

## Introduction

Parkinson’s disease (PD) is a progressive and ultimately devastating neurological disease. Development of new therapies is ongoing, and earlier stage interventions may show the most promise of slowing down the progression of the disease. Potential early-intervention therapies include gene transfer and deep brain stimulation (DBS) (1, 2), each requiring localization and surgical targeting of the subthalamic nucleus (STN).

Targeting of the STN using microelectrode mapping and macrostimulation in more advanced patients has been extensively performed in DBS for the treatment of advanced PD (3, 4). This widely accepted therapy is pursued when symptoms are not adequately controlled by medications (5, 6), and is often done later in the course of the disease (Hoehn–Yahr Stage III and IV). In these procedures, both microelectrode recording (MER) to identify the borders of the nucleus and macrostimulation to determine symptom reduction are used to locate the STN. There are uncertainties when applying these techniques to patients in earlier stages for early-interventional approaches. First, early in the course of the disease, MER characteristics are poorly understood, due in part to the paucity of data on the characteristics of the STN in a healthy human. Second, the utility of macrostimulation in early stages of the disease is not clear: in early-stage patients symptomatology is reduced and differences may not be clinically appreciable in the operative setting.

Thus, while locating STN in advanced disease patients is well understood and routinely performed at many centers in the context of DBS surgery, the ability to identify this nucleus in early-stage patients has not previously been explored in a sizeable cohort. Of particular surgical interest in this study was whether electrophysiological and macrostimulation mapping techniques established and refined in late PD populations could adequately describe and locate the STN in an earlier stage PD patient (Hoehn–Yahr Stage II) who will have substantially reduced symptomatology. The intraoperative MER technique and results reported here show that targeting can be successfully accomplished using traditional methods, even in patients with limited symptomatology, indicating feasibility of surgical targeting for early-stage interventions.

## Materials and Methods

### Patient selection

The methods reported here are drawn from an ongoing pilot clinical trial testing the safety and efficacy of DBS therapy in early-stage PD at Vanderbilt University Medical Center (NCT00282152, FDA investigational device exemption G050016). All procedures were in accordance with the ethical standards of Vanderbilt University Institutional Review Board (IRB approval 040797) and with the Helsinki Declaration of 1975 (rev 1983). Between August 2006 and April 2009, 30 subjects with early-stage PD were enrolled in a prospective, randomized, single-blind clinical trial comparing optimal drug therapy to bilateral STN stimulation plus optimal drug therapy (1, 7). Preselection criteria were patients aged 50–75 who had been on medication between 6 and 48 months, free of motor fluctuations such as levodopa associated dyskinesias or unexpected “on/off” phenomena. These patients were earlier stage than in a previous study of DBS in patients with early motor fluctuations (8). After informed consent was obtained, patients underwent a detailed screening evaluation to ensure their eligibility for the trial. Subjects were required to be Hoehn–Yahr Stage II in the “off” medication state and exhibit a 30% improvement in their UPDRS-III score between the “off” and “on” medication states (see baseline characteristics, Table 1). The screening also included a neuropsychological assessment, psychiatric evaluation, and brain MRI scan to identify abnormalities that would prevent the placement of DBS electrodes. Patients meeting inclusion criteria underwent 1 week of medication washout and baseline assessment and were randomized into two groups of 15 subjects (optimal drug therapy vs. DBS plus optimal drug therapy). Those randomized to receive DBS were implanted within 2 months of their baseline assessment, data presented here.

**Table 1:** **Baseline characteristics of patients [adapted with permission from Charles et al. (7), l-DOPA equivalency based on equation from Deusch et al. (9)]**.

Characteristic	*n* = 15
**GENDER**	
Male	14
Female	1
**AGE (YEARS)**	
Mean	60 ± 6.8
Range	52–74
**BASELINE MEDICINE USE**	
Mean duration (years)	2.2 ± 1.4
Mean l-DOPA equivalents (mg/day)	451 ± 304
**BASELINE UPDRS SCORE**	
Mean total	39 ± 14
Mean UPDRS-III	15 ± 8.5

### Presurgical image-based targeting

For patients randomized to the DBS group, stereotactic targeting and electrode insertion was performed using a rapid prototyped, miniature stereotactic system (WayPoint™ Stereotactic System; FHC Inc., Bowdoin, ME, USA) described in detail elsewhere (10). Preoperative MRI (standard T1 and T2 weighted) and CT imaging of the brain, in combination with an assessment of AC–PC coordinates, was used to target the dorsolateral STN (11, 12).

### Surgical methods

In preparation for implantation of the DBS leads, subjects discontinued dopamine agonist medications at least 48 h prior to surgery, and all other antiparkinsonian medications were discontinued 24 h prior to surgery (11). On the day of surgery, patients were brought to the operating room and placed in a semi-recumbent position. Under local anesthesia (lidocaine/marcaine) and with minimal IV sedation (dexmedetomidine/remifentanil), a burr hole and durotomy were performed to expose the brain surface. Using the miniature, rapid prototyped frame (microTargeting Platform™; FHC Inc., Bowdoin, ME, USA), one or two electrode microdrives were attached to the platform. IV sedation was discontinued at least 20 min before microelectrode mapping to ensure patients were alert and the STN fully responsive, fully consistent with established practices for later-stage DBS at Vanderbilt.

### Intraoperative mapping and targeting

Microelectrode recording was used to confirm STN location, and to overcome any intraoperative shift in target position due to cerebrospinal loss or edema (13–15). Arrays of 3–4 tungsten microelectrodes (0.3–1.0 MΩ at 1 kHz; Model 44970R; FHC Inc., Bowdoin, ME, USA) were attached to each microdrive via guide tubes spaced 2 mm apart in a “Ben-gun” configuration (10, 11). For 11 patients, neurophysiology was mapped using a four-channel Leadpoint recording system (Medtronic, Inc., Minneapolis, MN, USA). These MERs were band-pass-filtered (0.5–5 kHz), amplified, displayed, and digitally stored (24 kHz sampling rate). For these patients the mapping procedure was performed sequentially, first on one hemisphere and then repeated for the opposite hemisphere (left brain first in nine patients). For the remaining 4 patients, MER was obtained using an eight-channel Guideline 4000 system (FHC, Bowdoin, ME, USA). Signals were band-pass-filtered (0.5–5 kHz), amplified, displayed, and digitally stored (48 kHz sampling rate). For these patients both sides were able to be mapped simultaneously, a common practice at our center. Ten-second MERs were made at regular intervals (every 0.5 mm) along a pre-defined trajectory to the STN starting 10 mm above the preoperatively defined target and finishing 5–8 mm below target, usually a few millimeters inferior to the dorsal border of the SNr. Recordings within the thalamus, zona incerta (Zi), STN, and SNr were classified by an experienced neurophysiologist. STN traces were classified using accepted criteria, namely, increased background activity (neuronal “hash”) and high-rate irregularly firing neurons [e.g., Ref. (3, 4)], verified using offline analysis. Fifteen patients were randomized to the surgery group, but one patient’s electrophysiology was irrecoverable for *post hoc* analysis, so this report reflects neurophysiology from 14 patients.

After the MER mapping, macrostimulation mapping along the extent of electrophysiologically identified STN was then performed for all tracts under the supervision of a movement disorders neurologist to characterize the stimulation thresholds for reduction in rigidity (efficacy) and side effects (such as paresthesias, muscular contraction, or eye deviation). Rigidity and tremor were rated in a combined rating to be improved from 0 to 100%. Stimulation side effects (e.g., paresthesias and contraction) and affected body part (e.g., eyes and hand) were recorded. The range of current used for the efficacy and side effect mapping was approximately 0–5 mA, stepped by 0.5 mA.

After microelectrode mapping and macrostimulation effects were completed for all tracts, final target selection for locating the center of the quadripolar DBS lead (#3389; Medtronic Neuromodulation, Minneapolis, MN, USA) was determined by assessing the optimal results of three factors: MER definition of the dorsolateral border of STN, intensity of stimulation needed for reduction in rigidity of the contralateral upper extremity, and intensity of stimulation that resulted in undesirable side effects. The DBS lead was implanted in the tract that produced the best efficacy upon low threshold macrostimulation and the least side effects, where the two middle contacts spanned the most efficacious macrostimulation zone. Once the most favorable track and depth for stimulation were identified, the test electrode was replaced with a permanent DBS lead (Model 3389; Medtronic, Inc., Minneapolis, MN, USA), which was tested for functionality, affixed to the skull, coiled under the scalp, and prepared for connection to bilateral implantable pulse generators (IPG, Model 7426 Soletra Neurostimulator; Medtronic, Inc., Minneapolis, MN, USA) during a follow-up procedure within 10 days. The average location of the centroid of the lead was 10.7 mm lateral, 1.1 mm posterior, and 3 mm inferior to the mid-commissural point [see Ref. (11)]. Other than a longer time for medicine to be discontinued presurgery [see Ref. (5)], the details of this procedure do not differ significantly from the procedure used for DBS-implantation in later-stage PD patients at this center (10).

### Offline microelectrode recording analyses

In-house MATLAB scripts (MathWorks Inc., Natick, MA, USA) were used to calculate all electrophysiological measures; these were verified by Neuroexplorer 4 (Nex Technologies, Littleton, MA, USA) analyses. Tracks with <1.5 mm of STN recordings were excluded from analysis. Individual units were sorted from background using standard spike-sorting methods including automated cluster analysis of principal components (Spike Sorter v2.8, Plexon Inc., Dallas, TX, USA). As an aggregate and robust measure of neural activity, root mean square (RMS) measures of voltage were calculated from raw, unsorted data. For firing rate measures, only demonstrable single units were included in this analysis; poorly isolated units and multiple units with overlapping clusters in 2D PC space (MANCOVA *p* > 0.05) were excluded from further analysis.

## Results

**Figure 1:**
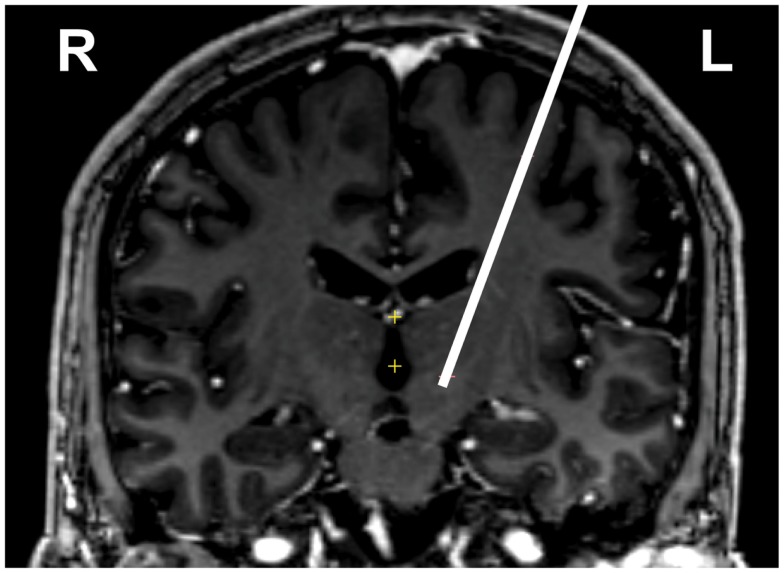
**Preoperative image guided targeting of left STN for one subject**. White line is the trajectory, shown on top of a coronal section at level of target, at bottom of line (actual tract is paracoronal). Image is T1 contrast weighted, neurological conventions where left is on image right.

Here we report the targeting and mapping procedure from DBS lead implantation in 15 patients with early PD. All subjects are Caucasian (one female), and the baseline UPDRS (part III) scores demonstrate only very mild symptomatology (see Table 1 for detailed description). Image-based targeting using preoperative CT and MRI allowed for approximate localization of the STN in each patient (Figure 1).

**Figure 2:**
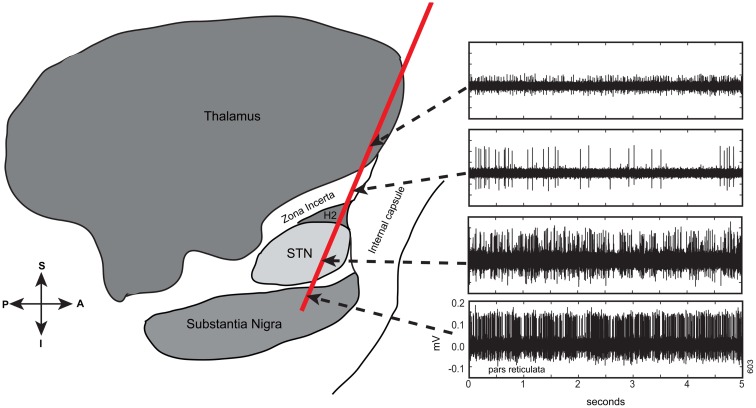
**Electrophysiological traces taken along a trajectory to and though the STN from a mapping pass of a single tract in early PD (one of four tracts)**. Ten-second long traces were taken but for ease of visualization of detail, the first 5 s are shown. *Y*-axes scaled identically to bottom panel. These traces are typical of what is encountered when passing from thalamus to Zi through STN to substantia nigra (pars reticulata). Note that the qualitative activity along the pass is not different from what is encountered when targeting during later-stage PD [e.g., Ref. (3)]. Pass superimposed on a sagittal schematic adapted from Schaltenbrand and Wharen (16).

**Figure 3:**
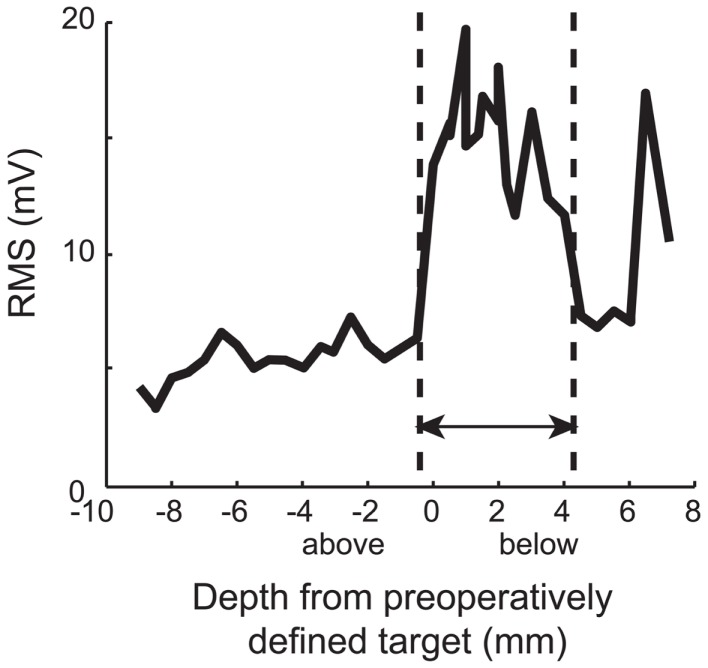
**Typical RMS by depth**. Note the strong increase in RMS along the extent that was identified as STN (dotted lines). In early PD, an increase in RMS is an indicator of STN location, much as it is in later stages. Zero indicates the level of target from preoperative plan. These data are derived from pass reflected in Figure 1.

Electrophysiology recorded during surgery revealed that at least one electrode track per hemisphere contained electrophysiologically identifiable STN (range: 1–4 per hemisphere). In total, 68 tracts that contained at least 1.5 mm of STN were identified in 28 hemispheres. In Figure 2, an exemplar pass of electrophysiological traces by structure is shown. These traces were similar to what is encountered in later-stage DBS surgery [see Ref. (3)]. Specifically, in a typical pass, we found that thalamic activity was characterized by low baseline activity, with well isolated action potentials. The Zi was characterized by sparse neural activity, but when cells were encountered, they had tonic firing patterns with high amplitude action potentials, well isolated from background. The STN, even in early Parkinson’s disease patients, was characterized by high baseline neuronal activity, with isolated neurons having high frequency and irregular firing rates. Even though these action potentials were also high amplitude, their relative amplitudes relative to baseline were lower than surrounding structures, a characteristic of STN. After 1–2 mm of low activity quiet zone, corresponding to white matter tracts, the substantia nigra (pars reticulata; SNr) was commonly encountered, a structure that exhibited high firing rates, with well isolated action potentials on top of relatively low baseline activity. Indeed, this pattern of results is confirmed when plotting RMS of voltage with depth, a measure easily accessible intraoperatively (Figure 3). Of note is the strong increase in RMS along the extent that was identified as STN (dotted lines).

**Figure 4:**
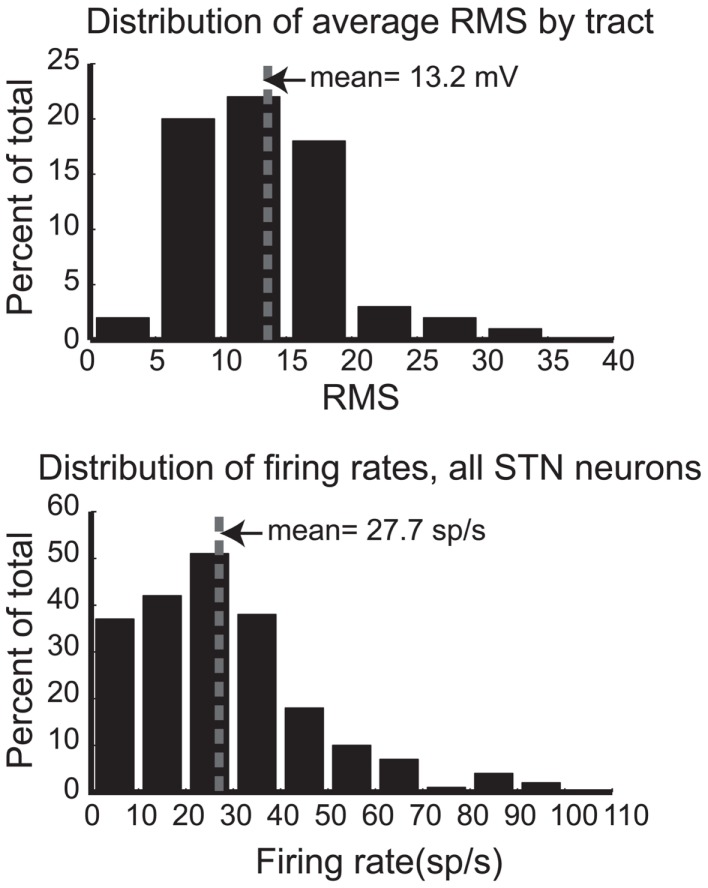
**Distributions of root mean square power (RMS, upper panel) for each STN pass and firing rates (lower panel) for all STN neurons**. Mean values are indicated by the dotted line.

To quantitatively describe the electrophysiological activity of the STN in these patients, Figure 4 (upper panel) shows the distribution of average RMS activity for all STN tracts across all hemispheres and patients. The mean RMS of 13.2 mV is indicated by a dotted line. A subset of this data was presented in an earlier report (17), and for ease of comparison the figures follow their conventions. Figure 4 (lower panel) shows the distribution of firing rates for all identified single units (*n* = 210) in STN from 13 hemispheres. Mean firing rate is 27.7 sp/s, indicated by the dotted line. These values are consistent with the earlier report and are lower than values of those later-stage patients.

After the dorsoventral extent of the STN was determined, efficacy and side effect profile of all electrode tracts was assayed through macrostimulation mapping. From 1 to 2 mm above the STN to the bottom of STN (where limiting side effects were elicited at very low currents), stimulation effects were determined, where the range of current was from 0 to 4 mA, stepped by approximately 0.5 mA. Rigidity and tremor improved from 0 to 100% (combined rating, see Materials and Methods) at thresholds from 0.5 to 4 mA. Stimulation side effects included facial and hand paresthesia, dysarthria, and gaze preference at thresholds from 1 to 4 mA.

Perioperative events are summarized in Table 2. Perioperative adverse events for this trial are similar to those already reported to be associated with DBS therapy for PD. One subject experienced a stroke, causing persistent but mild cognitive impairment and weakness in the right face and arm, which subsequently resolved over several months. This was the only perioperative event lasting longer than 3 months; the remaining 14 subjects experienced only typical post-operative discomfort and mild transient adverse effects that resolved in the 3-month perioperative period.

**Table 2:** **Perioperative adverse events, *n* = 56 [adapted with permission from Kahn et al. (11)]**.

Type of adverse event	Transient	Ongoing
**RELATED TO PROCEDURE OR DEVICE**		
Wound healing problems	10	0
Erythema	2	0
Edema	2	0
Pain	2	0
Drainage	2	0
Tingling	0	1
Tenderness	1	0
Headache	5	0
Edema	4	0
Scalp	2	0
Facial	2	0
Confusion	4	0
Imbalance	3	0
Drowsiness	2	0
Nausea	2	0
Vomiting	2	0
Expressive aphasia	2	0
Neck problems	2	0
Pain	1	0
Stiffness	1	0
Throat problems	2	0
Pain	1	0
Edema	1	0
Aborted procedure	2	0
Hematoma	1	0
Dysphagia	1	0
Intracranial edema	1	0
Basal ganglia infarct	0	1
Extremity weakness	1	0
Hallucination	1	0
Urinary retention	1	0
Constipation	0	1
Rigidity	1	0
Divergent gaze	1	0
Apnea	1	0
**RELATED TO STUDY**		
Syncope	1	0
**NOT RELATED TO STUDY**		
Incidental CT imaging sinus findings	0	4
Paresthesias	1	0
Fever	1	0
Chest soreness	1	0

*The “ongoing” column indicates any symptoms lasting longer than 3 months*.

## Discussion

Here we report targeting of the STN and surrounding structures in an ongoing prospective trial testing STN-DBS in patients with early-stage PD. The results reported here suggest that the careful application of accepted surgical methods including image guided targeting, microelectrode mapping, and macrostimulation can be used to successfully target the STN in DBS-implantation procedures for early-stage PD, even in patients with minimal symptomatology. Consistent for all traces recorded from these patients, the qualitative activity along the pass is not different from what is commonly encountered when targeting the STN during later-stage PD (3, 4). In previous studies of a subset of these patients, the measured STN firing rates and RMS have been found to be slightly lower than in later-stage PD patients (17), presenting a potential difficulty for surgical targeting. However, in this paper we report the changes from the white matter and surrounding structures are still sufficiently robust enough for accurate targeting. In early PD, much as it does in later stages, an increase in global neural activity measures (RMS) is an indicator of STN location, much as it is in later-stage PD. Though more research is needed, this increase in STN activity is most likely due to disease progression rather than age *per se*. Strikingly, even with mild symptoms associated with early stages of the disease, dopamimetic effects of stimulation could still be elicited to confirm the most efficacious portion of the STN.

While presenting the targeting methods used at this center, these data also provide a foundation upon which future clinical trials can be designed for early-intervention therapies. One limitation of comparing data from multiple centers is that DBS-implantation centers utilize many different techniques for intraoperative recordings when attempting to identify the optimal target for implantation. Toward this end, this reports a detailed description of both the intraoperative technique for data collection, as well as the method for recording analysis leading to our findings. These subjects demonstrate a robust physiology that allowed traditional MER and stimulation techniques to be used in the effective surgical targeting of the STN, indicating feasibility of the surgical approach in proposed early-stage interventions targeting the STN (1, 2), which require surgical targeting of the STN. As surgical techniques are improved and risks reduced, is hoped that early-interventional therapies, including those targeting the STN, show promise in slowing the progressive motor and cognitive decline in PD.

## Author Contributions

Corrie R. Camalier analyzed data, wrote, and revised manuscript. Peter E. Konrad contributed to conception and study design, collected data, reviewed and critiqued manuscript, and provided supervision. Chandler E. Gill contributed to study conception and design, collected data, and reviewed and critiqued manuscript. Chris Kao collected data and reviewed and critiqued manuscript. Michael R. Remple analyzed data and reviewed and critiqued manuscript. Hana M. Nasr analyzed data and provided technical support. Thomas L. Davis, Peter Hedera, and Fenna T. Phibbs contributed to conception and study design and reviewed and critiqued manuscript. Anna L. Molinari collected data, wrote portions and reviewed and critiqued manuscript, and provided technical support. Joseph S. Neimat contributed to conception and study design and critiqued the manuscript. David Charles contributed to conception and study design, collected data, wrote portions, revised and critiqued manuscript, obtained funding, and provided supervision.

## Conflict of Interest Statement

We wish to include the following disclosures: Corrie R. Camalier, Chris Kao, and Michael R. Remple receive partial salary support from Sentient Medical Services. Peter E. Konrad receives research funding by Medtronic and the NIH, is on the speaker’s bureau for Medtronic and FHC, and also holds a fiduciary position (Board of Directors) with Neurotargeting, the American Society for Stereotactic and Functional Neurosurgery, and the North American Neuromodulation Society. Fenna T. Phibbs has done consulting work for Medtronic and has received speaking honoraria from Teva. Peter Hedera has received speaking honoraria from Teva. Joseph S. Neimat has done consulting work for Medtronic and has received research funding from Medtronic and the NIH. Vanderbilt University has received income in excess of $10,000 from grants or contracts with Medtronic, Allergan, Ipsen, Merz, UCB, and Teva for educational or research programs led by David Charles. David Charles receives income in excess of $10,000 from Medtronic, Allergan, Ipsen, and the Alliance for Patient Access for education and consulting services. Chandler E. Gill, Hana M. Nasr, Thomas L. Davis, and Anna L. Molinari do not have conflicts of interest.
